# Biotransformation of Sophorabioside in the Fruits and Small Branches of *Sophora japonica* into Sophoricoside Using α-L-Rhamnosidase from *Chloroflexus aurantiacus*

**DOI:** 10.4014/jmb.2505.05034

**Published:** 2025-08-07

**Authors:** Su-Hwan Kang, Yeong-Su Kim, Hwan Lee, Kyung-Chul Shin

**Affiliations:** 1 Department of Bioscience and Biotechnology, Konkuk University, Seoul 05029, Republic of Korea; 2 R&D Center, Ildusanbang Co., Cheongsong 37434, Republic of Korea; 3 R&D Center, Bebornnature Co., Cheonan 31154, Republic of Korea; 4 Sphere Corporation, Seoul 06159, Republic of Korea; 5 Department of Bioscience and Biotechnology, Hankuk University of Foreign Studies, Yongin 17035, Republic of Korea

**Keywords:** *Sophora japonica* extract, bioactive flavonoids, sophorabioside, sophoricoside, enzymatic conversion, anti-inflammatory activity

## Abstract

*Sophora japonica* L., a medicinal herb used in East Asia, contains bioactive flavonoids such as sophoricoside and sophorabioside. Sophoricoside, a more bioavailable and therapeutically potent form, is known for its pharmacological properties, including anti-inflammatory, -cancer, and -osteoporotic effects. However, conversion of sophorabioside into sophoricoside has not been extensively studied. In this study, we aimed for the enzymatic conversion of sophorabioside, abundant in the *S. japonica* fruits and small branches, into sophoricoside using α-L-rhamnosidase from *Chloroflexus aurantiacus*. Optimal reaction conditions for this biotransformation were established, with maximal enzymatic activity observed at pH 6.0 and 55°C. Substrate specificity analysis revealed high selectivity of the enzyme for neohesperidose-containing flavonoids, including sophorabioside. Sophoricoside was efficiently produced from *S. japonica* fruit and branch extracts, enhanced by 2.5% methanol. The biotransformed extracts demonstrated significant lipoxygenase inhibitory activity, suggesting enhanced anti-inflammatory potential, highlighting the potential of *S. japonica*-derived sophoricoside-enriched products as functional food supplements with improved therapeutic properties.

## Introduction

*Sophora japonica* L., in the Leguminosae family, is distributed in China, Japan, and South Korea. It is consumed as food and is a traditional medicinal herb [1, 2]. Fructus sophorae, the dried ripe *S. japonica* fruit, exhibits anti-angiogenic [3], -tumorigenic [4], -osteoporotic [5], -oxidant [6], and -atherosclerosis [7] activities due to its bioactive compounds, including alkaloids, amino acids, fatty acids, triterpenes, phospholipids, rutin, quercetin, genistein, and sophoricoside [8-10].

Sophoricoside (genistein-4'-*O*-glucoside) is an abundant flavone in Fructus sophorae that can be used to treat osteoporosis in postmenopausal women [11]. Furthermore, sophoricoside exhibits anti-cancer, -inflammatory, -oxidation, -obesity, and -diabetic, immunosuppressive, estrogenic, stimulation of osteoblast proliferation, and immunomodulatory effects [12-16]. Fructus sophorae contains sophorabioside (genistein-4'-*O*-neohesperidoside), a rhamnoside form of sophoricoside in concentrations as high as sophoricoside [17]. Additionally, small branches of *S. japonica* contain sophorabioside but not sophoricoside [18]. Therefore, studies on the rhamnose hydrolysis of sophorabioside are required to increase the bioactive sophoricoside content in Fructus sophorae and to produce sophoricoside using waste branches.

Sophoricoside is a more bioavailable and therapeutically potent form; however, to our knowledge, no studies have sufficiently reported on the conversion of sophorabioside to sophoricoside. Previous studies have converted sophoricoside into genistein using β-glucosidase [19], microbial cells [20-22], and acid hydrolysis [23]. Among these methods, enzymatic biotransformation can provide superior selectivity and specificity and the highest productivity. In our previous study, α-L-rhamnosidase from *Chloroflexus aurantiacus* exhibited high activity for flavonoids such as rutin, hesperidin, and naringin [24], suggesting that the enzyme may catalyze the conversion of sophorabioside to sophoricoside.

In this study, we aimed to investigate the hydrolytic activity for rhamnose-containing flavonoids, including sophorabioside, using α-L-rhamnosidase from *C. aurantiacus*. The reaction conditions were optimized for the biotransformation of sophorabioside to sophoricoside, and sophoricoside was produced from sophorabioside extracted from the fruits and small branches of *S. japonica* using α-L-rhamnosidase from *C. aurantiacus*. After the biotransformation, anti-inflammatory activity was investigated for the application of sophorabioside-enriched fruits and small branches of *S. japonica* as improved functional food supplements.

## Materials and Methods

### Materials

The flavonoid standards hesperidin, narirutin, rutin, kaempferol 3-*O*-rutinoside, naringin, prunin, sophoricoside, neohesperidin, quercetin-3-*O*-neohesperidoside, kaempferol-3-*O*-neohesperidoside, sophorabioside, hesperetin-7-*O*-glucoside, isoquercitrin, and kaempferol 3-*O*-glucoside were purchased from Sigma-Aldrich (USA) and Chemfaces (China). The *S. japonica* fruits and small branches were obtained from Baekdudaegan National Arboretum, Bonghwa, Republic of Korea.

### Preparation of Extracts from *S. japonica* Fruits and Small Branches

The *S. japonica* fruit extract (SJFE) and small branch extract (SJSBE) were prepared using a previous extraction method [17, 18]. To extract flavonoids such as sophorabioside and sophoricoside, 100 g of dried fruit or small branch powder was added to 1 liter of 95% ethanol and sonicated thrice at 45°C for 3 h each time. After cooling, the precipitates were removed by filtering the mixture through a 0.45-μm filter, the ethanol was removed by evaporation, and then the residue was dissolved in 1 liter of distilled water. The solution was adsorbed onto a glass column (length × diameter: 700 × 41.4 mm) packed with Diaion HP20 resin (Mitsubishi Chemical Corporation, Japan) to prevent enzyme inhibition by free sugars. The column was eluted with 2 liters of distilled water to remove the unbound free sugars and other hydrophilic compounds leaving flavonoids attached to the resin. The adsorbed flavonoids were subsequently eluted with 2 liters of ethanol at 0.5 ml/min flow rate. The ethanol in the eluent was removed by evaporation, and the dried residue was dissolved in 1 liter of distilled water. The resulting sugar-free extracts (10%, w/v) were used for the conversion of sophorabioside into sophoricoside using α-L-rhamnosidase from *C. aurantiacus*.

### Gene Cloning and Culture Conditions for Enzyme Expression

*C. aurantiacus* DSM636 (DSMZ, Germany), *Escherichia coli* BL21 (New England Biolabs, UK), and pET-28a(+) vector (Novagen, Japan) were used as the sources for the DNA template of the α-L-rhamnosidase gene (GenBank Accession No. A9WDK5), host cells, and expression vector, respectively. The α-L-rhamnosidase gene from *C. aurantiacus* was cloned as described previously [24]. Recombinant *E. coli* BL21 expressing α-L-rhamnosidase from *C. aurantiacus* was cultured at 37°C in a 2-liter flask containing 500 ml Luria–Bertani medium and 20 μg/ml kanamycin at 37°C with agitation at 200 rpm. When the optical density at 600 nm was 0.8, 100 μM isopropyl-β-D-thiogalactopyranoside was added to the broth to induce the expression of α-L-rhamnosidase. The recombinant cells were subsequently cultured at 16°C with agitation at 150 rpm for a further 16 h to express the enzyme.

### Enzyme Preparation

Recombinant *E. coli* expressing α-L-rhamnosidase from *C. aurantiacus* was harvested from culture broth by centrifugation at 6,000 × g at 4°C for 25 min, washed with 0.85% NaCl and suspended in 50 mM sodium/phosphate buffer (pH 7.0) containing 300 mM NaCl and 5 mM imidazole with 1 mg/ml lysozyme. The suspended cells were disrupted by sonication on ice for 30 min. The cell debris and unbroken cells were removed by centrifugation at 13,000 ×*g* at 4°C for 20 min, and the obtained supernatant was applied to a Histrap HP affinity column (Thermo Fisher Scientific) equilibrated with 50 mM sodium/phosphate buffer (pH 7.0) on a fast protein liquid chromatography system. The bound recombinant enzyme was subsequently eluted with the same buffer containing a linear gradient of imidazole (10–250 mM) at 1 ml/min. The collected active fractions were dialyzed against 50 mM citrate/phosphate buffer (pH 6.0) at 4°C for 16 h. The resulting solution was used as a purified α-L-rhamnosidase.

### Hydrolytic Activity

Unless otherwise specified, the reaction was performed in 50 mM citrate/phosphate buffer (pH 6.0) containing 0.4 mM flavonoids, 7.424 U/ml of α-L-rhamnosidase, and 2.5% dimethyl sulfoxide (DMSO) at 55°C for 30 min. One unit (U) of enzyme activity for flavonoid was defined as the amount of enzyme required to liberate 1 μmol of sophoricoside from sophorabioside per minute at pH 6.0 and 55°C. The specific activity (U/mg) was defined as the amounts of flavonoids produced, such as hesperetin-7-*O*-glucoside, prunin, isoquercetin, kaempferol 3-*O*-glucoside, and sophoricoside as products per enzyme amount per unit reaction time. The amounts of flavonoids were determined using high performance liquid chromatography (HPLC) assay.

### Effects of Environmental Conditions

Temperature was varied from 40 to 70°C at pH 6.0 and pH values were varied from 5.0 to 7.0 at 55°C to estimate the effects of temperature and pH on the hydrolytic activity of α-L-rhamnosidase from *C. aurantiacus* for sophorabioside. The reactions were performed in 50 mM citrate/phosphate buffer containing 0.5 mM sophorabioside, 7.424 U/ml enzyme, and 10% (v/v) DMSO for 30 min. The effect of temperature on enzyme stability was monitored as a function of incubation time by placing the enzyme solution in 50 mM citrate/phosphate buffer (pH 6.0) at five different temperatures (45, 50, 55, 60, and 65°C). Samples were withdrawn at different time intervals and assayed. The experimental data for enzyme inactivation were fitted to a first-order curve. The rate constant (k_d_, min^−1^) was determined from the slope of the deactivation time course using the equation: ln(E_t_/E_0_) = −k_d_t. where E_t_ and E_0_ are the residual enzyme activity after heat treatment for time (t) and the initial enzyme activity before heat treatment, respectively. The half-life of thermal deactivation (t_1/2_) was calculated according to the following equation: t_1/2_ = ln(2)/k_d_.

Several organic solvents at concentrations of 5% and 10% (v/v), including ethanol, methanol, 3-methyl-1-butanol, isopropanol, formic acid, acetone, and DMSO, were tested for subsequent use in selecting the optimum solvent. The effect of solvent concentration on α-L-rhamnosidase activity was estimated by varying the concentration of ethanol, methanol, or DMSO from 1% to 10% (v/v). The reactions were performed in 50 mM citrate/phosphate buffer (pH 6.0) containing 0.5 mM sophorabioside, 7.424 U/ml enzyme, and solvent at 55°C for 30 min.

### Biotransformation of Sophorabioside into Sophoricoside

The optimal enzyme concentrations for producing sophoricoside from sophorabioside in SJFE and SJSBE were determined by varying the concentration of α-L-rhamnosidase from *C. aurantiacus* from 0.928 to 8.352 U/ml with 10% (w/v) SJFE and SJSBE and 2.5% methanol in 50 mM citrate/phosphate buffer (pH 6.0) at 55°C for 30 min. The optimal substrate concentrations for producing sophoricoside from sophorabioside in SJFE and SJSBE were determined by varying sophorabioside concentrations in SJFE and SJSBE from 0.045 to 0.54 g/l and from 0.086 to 1.03 g/l, respectively, with 5.568 U/ml α-L-rhamnosidase from *C. aurantiacus* and 2.5% methanol in 50 mM citrate/phosphate buffer (pH 6.0) at 55°C for 30 min. The time-course reactions for producing sophoricoside from sophorabioside in SJFE and SJSBE were investigated in 50 mM citrate/phosphate buffer (pH 6.0) containing 4.64 U/ml enzyme and 0.45 g/l sophorabioside in SJFE for 90 min, and 11.136 U/ml enzyme and 0.86 g/l sophorabioside in SJSBE, respectively, with 2.5% methanol at 55°C.

### LOX Inhibitory Assay

The lipoxygenase (LOX) inhibitory activity of the biotransformed SJFE and SJSBE for presenting anti-inflammatory activity was measured using a LOX inhibitor screening assay kit (Cayman Chemical, USA) at 4 μM of each sample. For all extract samples, the sum of sophorabioside and sophoricoside contained therein was added at 4 μM. Nordihydroguaiaretic acid (NDGA) as a standard LOX inhibitory chemical and baicalein (5,6,7-trihydroxyflavone) as an anti-inflammatory agent were used as positive controls. Test samples and positive controls were dissolved in methanol. Next, 90 μl 15S-LOX and 10 μl test samples were placed in the testing wells, and the reactions were initiated by adding 10 μl arachidonic acid to each well. All mixtures were incubated in a shaker and thoroughly mixed for 5 min, and 100 μl chromogen was then added to the wells to terminate the enzyme reaction. After the reaction, the hydroperoxide level produced from arachidonic acid by 15S-LOX was determined by measuring the UV absorbance at 500 nm. The LOX inhibitory activity (%) was calculated using the following formula: (C − T)/ C × 100, where C and T indicate the values of the UV absorbance at 500 nm without and with the test sample, respectively.

### HPLC Analysis

*n*-Butanol was added to the reaction solution at the same volume to stop the reaction and extract the substrate and product, and the mixture was separated into *n*-butanol and water fractions. The *n*-butanol of the collected *n*-butanol fraction was evaporated until completely dry, and methanol was added to the dried residue. Substrate and product in methanol were analyzed using an HPLC system (Agilent 1260, USA) equipped with a UV detector at 205 nm and a reversed phase AccQ-Tag column (150 × 3.9 mm, Waters, USA). The column was eluted at 30°C with a gradient of solvent I (0.1% trifluoroacetic acid and 99.9% acetonitrile, v/v) and solvent II (0.1% trifluoroacetic acid, 99.9% distilled water, v/v) of 10:90–40:60 for 40 min and 40:60–90:10 for 5 min with the flow rate fixed at 1.0 ml/min. The substrates hesperidin, narirutin, rutin, kaempferol-3-*O*-rutinoside, neohesperidin, naringin, quercetin-3-*O*-neohesperidoside, kaempferol-3-*O*-neohesperidoside, and sophorabioside were detected with retention times of 9.6, 8.2, 7.3, 8.5, 10.4, 8.9, 6.5, 7.8, and 9.9 min, respectively. The products hesperetin-7-*O*-glucoside, prunin, isoquercetin, kaempferol-3-*O*-glucoside, and sophoricoside were detected with retention times of 10.1, 8.5, 7.7, 9.6, and 9.3 min, respectively. The flavonoids in the reaction samples converted from different substrates were identified as having the same retention times along with the flavonoid standards.

## Results and Discussion

### Effects of pH and Temperature on the Biotransformation of Sophorabioside into Sophoricoside Using α-L-Rhamnosidase from *C. aurantiacus*

**Fig. 1:**
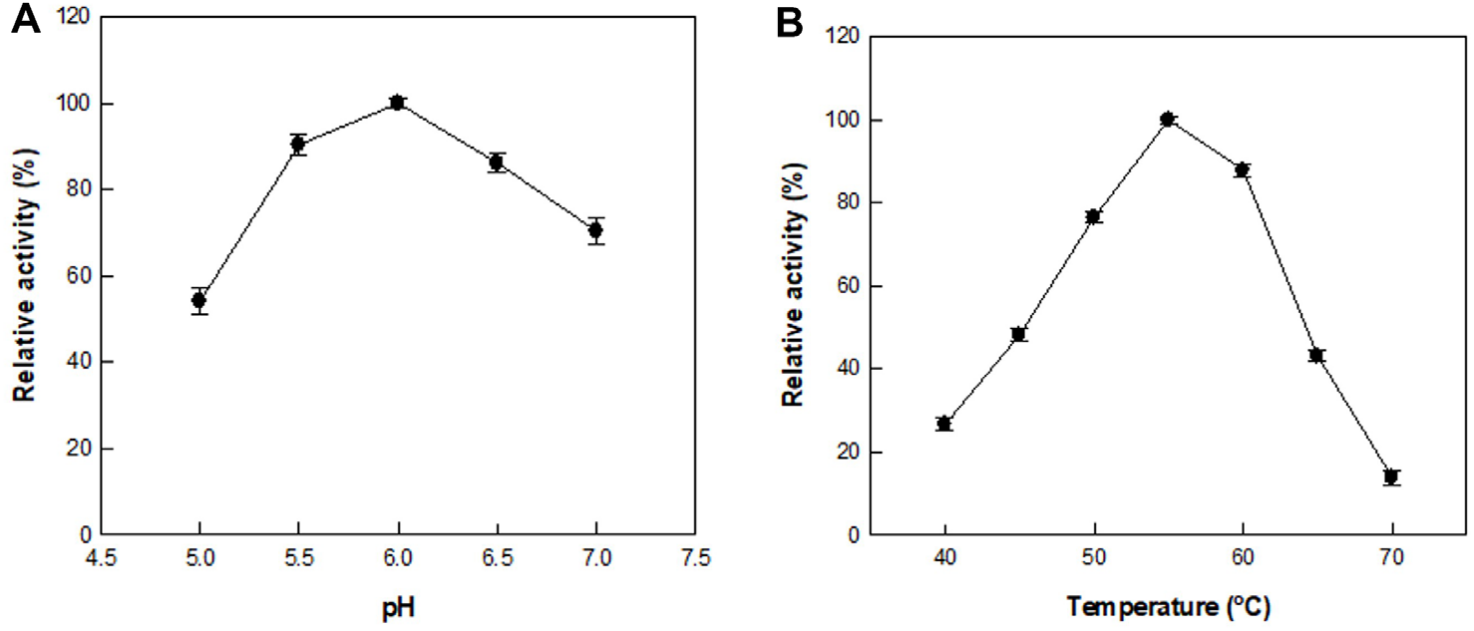
Effects of (A) pH and (B) temperature on the hydrolytic activity of α-L-rhamnosidase from *C. aurantiacus* mediating the conversion of sophorabioside to sophoricoside. Data represent the means of three experiments and error bars represent the standard deviations.

The effect of pH on hydrolytic activity of α-L-rhamnosidase from *C. aurantiacus* mediating the conversion of sophorabioside to sophoricoside was examined at pH ranging from 5.0 to 7.0 (Fig. 1A). Maximal activity was observed at pH 6.0, and the activity at pH 5.0 was approximately 50% of the maximum. The temperature was varied between 40 and 70°C to investigate its effect on the hydrolytic activity of α-L-rhamnosidase from *C. aurantiacus* mediating the conversion of sophorabioside to sophoricoside, and the activity was maximal at 55°C (Fig. 1B). The pH and temperature ranges for naringin and hesperidin hydrolysis by other hydrolytic enzymes were 4.0–6.0 and 37–60°C, respectively [25-29].

**Fig. 2:**
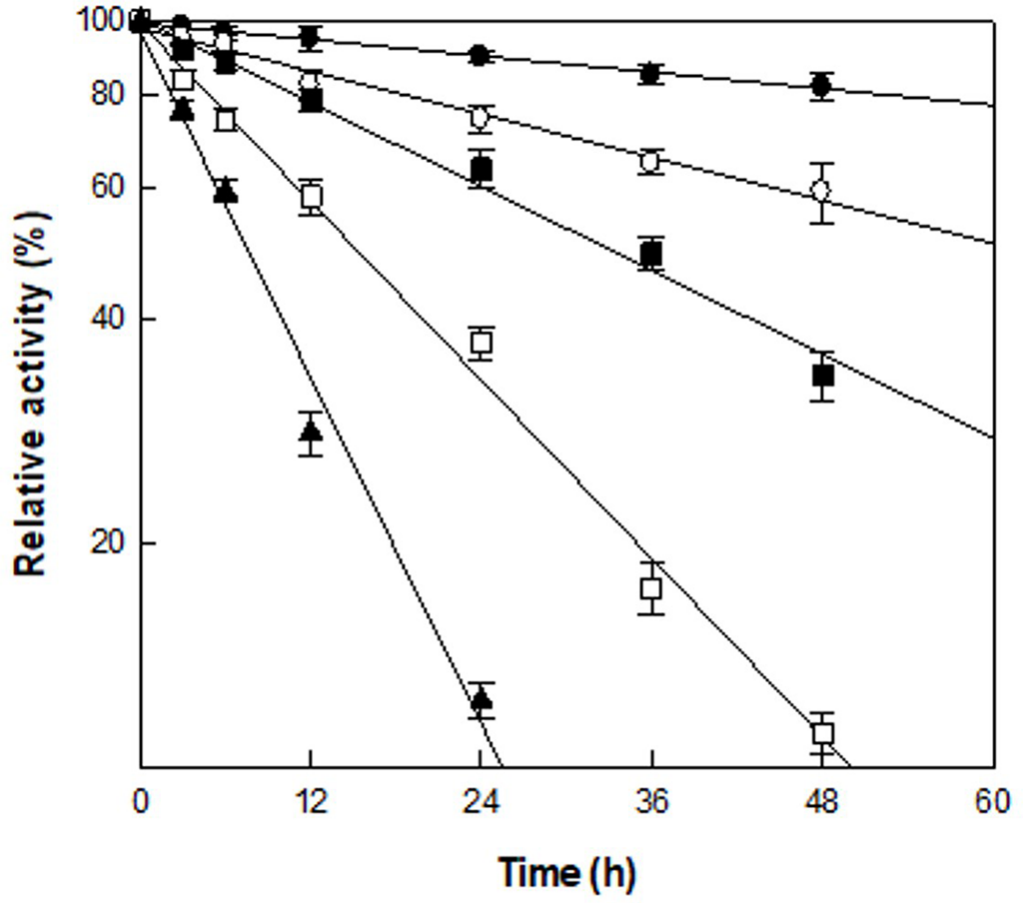
Effect of temperature on the enzyme stability. (●), (○), (■), (□), and (▲) represent 45, 50, 55, 60, and 65°C enzyme incubation temperatures, respectively, for various incubation times. A sample was withdrawn at each time interval, and the relative activity was determined. Relative activity was calculated by setting the enzyme activity at time zero as 100%. Data represent the means of three experiments and error bars represent the standard deviations.

The thermal stability of α-L-rhamnosidase from *C. aurantiacus* was examined within the temperature range of 45–65°C (Fig. 2). The half-lives of the enzyme were 164.9, 62.7, 32.2, 15.1, and 7.8 h at 45, 50, 55, 60, and 65°C, respectively. The thermal deactivation rate constant k_d_ (min^−1^) at 45 and 50°C was approximately 0.2- and 0.5-fold slower than that at 55°C, respectively (Table 1). However, the rates at 60 and 65°C were 2.1- and 4.1-fold slower than that at 55°C, respectively. Therefore, 55°C was an appropriate temperature for the hydrolysis of sophorabioside to sophoricoside by α-L-rhamnosidase from *C. aurantiacus*.

### Effect of Solvent on the Biotransformation of Sophorabioside into Sophoricoside by α-L-Rhamnosidase from *C. aurantiacus*

**Fig. 3:**
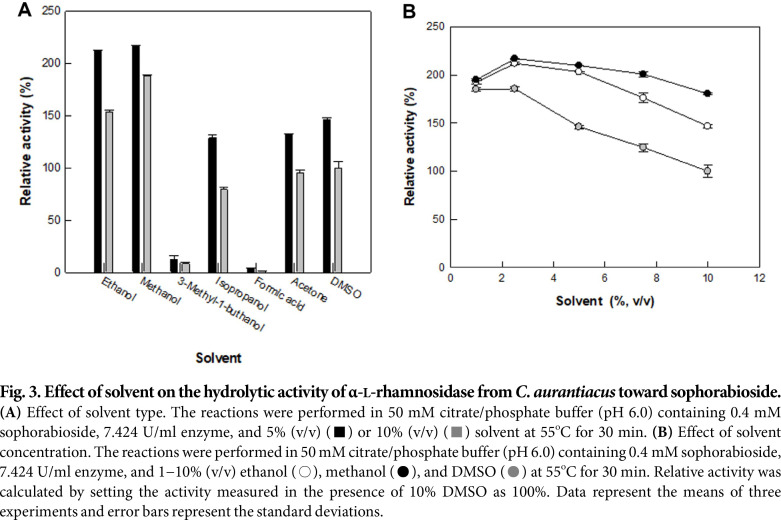
Fig. 3

The effect of organic solvent on the hydrolytic activity of α-L-rhamnosidase from *C. aurantiacus* mediating the conversion of sophorabioside to sophoricoside was examined at 5% and 10% (v/v). Among the solvents tested, ethanol and methanol were most effective for the hydrolytic activity (Fig. 3A). The effect of varying ethanol or methanol concentrations from 0.5% to 10% (v/v) with DMSO as a control on the sophorabioside hydrolytic activity during conversion to sophoricoside was evaluated. The activity using methanol was the highest in all concentrations, and it was 2.2-fold higher than that using 10% (v/v) DMSO at 2.5% (v/v) (Fig. 3B). Therefore, the enzyme activity was determined with the addition of 2.5% (v/v) methanol.

### Substrate Specificity of α-L-Rhamnosidase from *C. aurantiacus* for Flavonoids

**Fig. 4:**
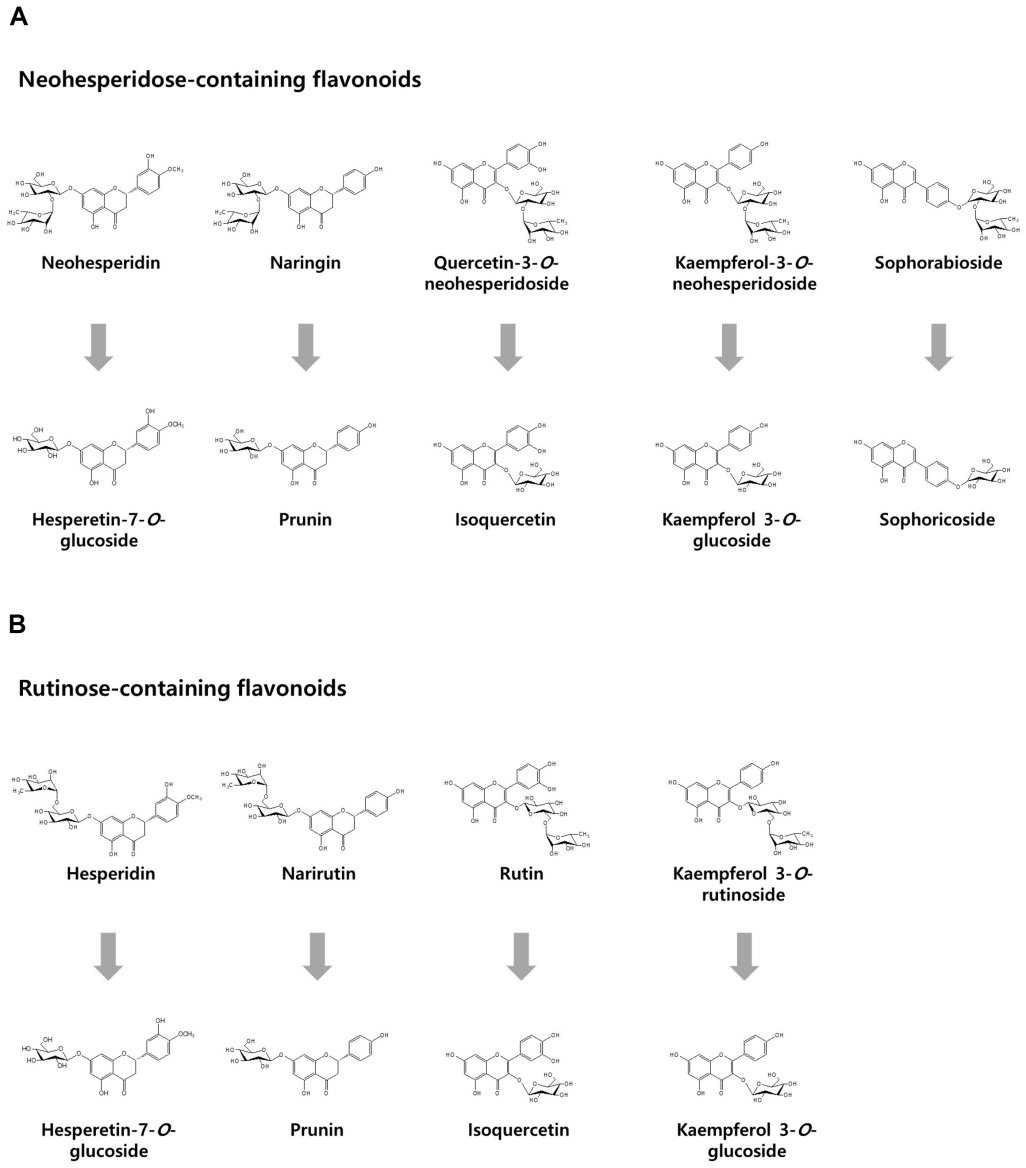
Conversion of rhamnose-containing flavonoids by α-L-rhamnosidase from *C. aurantiacus*. (**A**) Conversion of neohesperidose-containing flavonoids such as neohesperidin, naringin, quercetin-3-*O*-neohesperidoside, kaempferol-3-Oneohesperidoside, and sophorabioside into hesperetin-7-*O*-glucoside, prunin, isoquercetin, kaempferol-3-*O*-glucoside, and sophoricoside, respectively. (**B**) Conversion of rutinose-containing flavonoids such as hesperidin, narirutin, rutin, and kaempferol into hesperetin-7-*O*-glucoside, prunin, isoquercetin, kaempferol-3-*O*-glucoside, respectively.

Fig. 4 illustrates the enzymatic conversion of rhamnose-containing flavonoids by *C. aurantiacus* α-L-rhamnosidase, comparing substrates with either neohesperidose or rutinose sugar moieties. To evaluate the enzyme’s substrate specificity, we tested a set of flavonoids including neohesperidin, naringin, quercetin-3-*O*-neohesperidoside, kaempferol-3-*O*-neohesperidoside, and sophorabioside (neohesperidose-containing), as well as hesperidin, narirutin, rutin, and kaempferol-3-*O*-rutinoside (rutinose-containing). The enzyme exhibited notably higher activity toward the neohesperidose group, particularly sophorabioside and kaempferol-3-*O*-neohesperidoside, compared to the rutinose group. In contrast, no activity was observed toward the corresponding glucoside forms of these flavonoids, as previously reported [24]. These results suggest that both the disaccharide type and the glycosylation position plays critical roles in substrate recognition. Full activity values are provided in Table S1.

### Effects of Enzyme and Substrate Concentrations on the Production of Sophoricoside from Sophorabioside in *S. japonica* Fruit and Small Branch Extracts

**Fig. 5:**
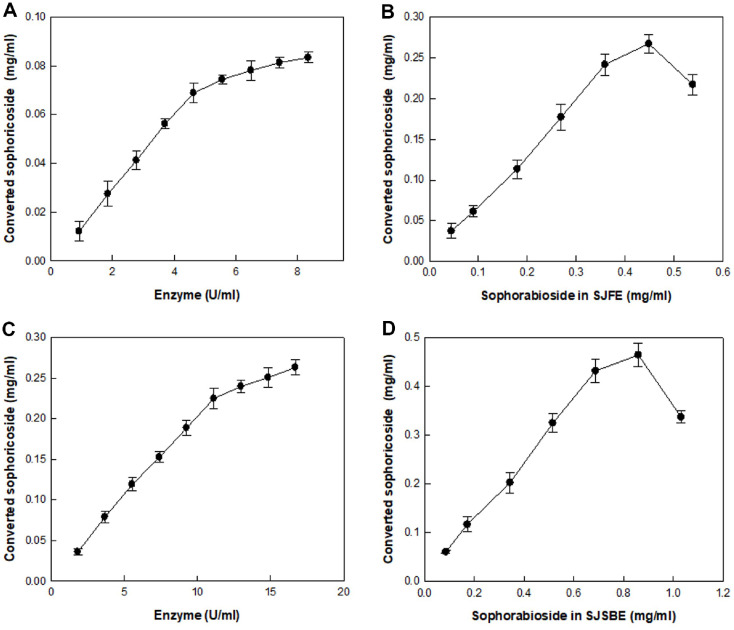
Effects of enzyme and substrate concentrations on the production of sophoricoside from sophorabioside in SJFE and SJSBE. (**A**) Effect of enzyme concentration on the production of sophoricoside from sophorabioside in SJFE. (**B**) Effects of substrate concentration on the production of sophoricoside from sophorabioside in SJFE. (**C**) Effect of enzyme concentration on the production of sophoricoside from sophorabioside in SJSBE. (**D**) Effects of substrate concentration on the production of sophoricoside from sophorabioside in SJSBE. Data represent the means of three experiments and error bars represent the standard deviations.

The sophorabioside and sophoricoside contents in SJFE were 0.9 mg/g and 0.4 mg/g, respectively, which were 1.4- and 1.1-fold higher than those observed in previous studies [17, 18]. Sphorabioside and sophoricoside contents in SJSBE were 1.7 and 2.8 mg/g, respectively, which was considerably different from that observed in a previous study in which SJSBE contained only sophorabioside (0.26 mg/g) [18]. The effect of enzyme concentration on the production of sophoricoside from sophorabioside in SJFE and SJSBE was investigated using 0.09 and 0.17 mg/ml sophorabioside in 10% (w/v) SJFE and SJSBE, respectively, as substrates and varying the enzyme concentration from 0.928 to 8.352 U/ml and 1.856 to 16.704 U/ml, respectively, for 30 min (Fig. 5A and 5C). The production of sophoricoside from sophorabioside in SJFE and SJSBE gradually increased with increase in enzyme concentration. However, increasing the enzyme concentration >4.64 U/ml and >11.136 U/ml for SJFE and SJSBE, respectively, decreased the sophoricoside production rate from sophorabioside, indicating that the optimum enzyme concentrations were 4.64 and 11.136 U/ml for SJFE and SJSBE, respectively. Under these enzyme concentrations, the effect of substrate concentration was investigated by varying sophorabioside concentrations in SJFE and SJSBE from 0.045 to 0.54 mg/ml and from 0.086 to 1.03 mg/ml, respectively, for 30 min (Fig. 5B and 5D). Converted sophoricoside increased with increasing concentrations of sophorabioside in SJFE and SJSBE to 0.45 and 0.86 mg/ml, respectively. Therefore, 0.45 and 0.86 mg/ml sophorabioside in SJFE and SJSBE, respectively, were the optimum substrate concentrations for sophoricoside production.

The time-course reactions for producing sophoricoside from sophorabioside in SJFE and SJSBE were investigated in 50 mM citrate/phosphate buffer (pH 6.0) containing 4.64 U/ml enzyme and 0.45 g/l sophorabioside in SJFE for 90 min, and 11.136 U/ml enzyme and 0.86 g/l sophorabioside in SJSBE, respectively, with 2.5% methanol at 55°C.

### Sophoricoside Production from *S. japonica* Fruit and Small Branch Extracts by α-L-Rhamnosidase from *C. aurantiacus*

**Fig. 6:**
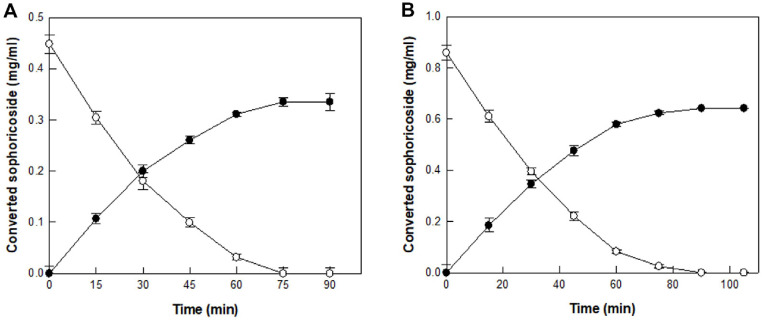
Time-course reactions on the production of sophoricoside (●) from sophorabioside (○) in SJFE and SJSBE by α-L-rhamnosidase from *C. aurantiacus*. (**A**) Time-course reactions on the production of sophoricoside from sophorabioside in SJFE. (**B**) Time-course reactions on the production of sophoricoside from sophorabioside in SJSBE. Data represent the means of three experiments and error bars represent the standard deviations.

The time-course reactions for producing sophoricoside from sophorabioside in SJFE or SJSBE were carried out under the optimized conditions of pH 6.0, 55°C, 2.5% methanol, 4.64 or 11.136 U/ml α-L-rhamnosidase from *C. aurantiacus*, and 0.45 or 0.86 g/l sophorabioside, respectively. Sophoricoside was produced from sophorabioside in SJFE and SJSBE with concentrations of 0.34 and 0.64 mg/ml and volumetric productivities of 0.27 and 0.43 mg/l/h for 1.25 and 1.5 h, respectively (Fig. 6). All sophorabioside in SJFE and SJSBE were completely converted to sophoricoside by α-L-rhamnosidase from *C. aurantiacus* with a molar yield of 100%. To the best of our knowledge, this is the first report of sophoricoside production from sophorabioside, which considerably increased sophoricoside content in *S. japonica* extracts.

### Anti-Inflammatory Activity of Biotransformed *S. japonica* Fruit and Small Branch Extracts

The *in vitro* anti-inflammatory activity of biotransformed SJFE and SJSBE was evaluated using a LOX inhibitory assay (Table 2). The LOX inhibitory values of NDGA and baicalein as positive controls were 67.25 and 3.18%, respectively. The LOX inhibitory values of SJFE (29.52%) and SJSBE (38.30%) were higher than that of sophorabioside (22.08%) because they contained sophoricoside. The LOX inhibitory values of sophoricoside and biotransformed SJFE and SJSBE were 49.09%, 51.85%, and 53.09%, respectively, which were 2.22-, 1.76-, and 1.39-fold higher than those of sophorabioside, SJFE, and SJSBE, respectively. The biotransformation of SJSBE exhibited a lesser change in anti-inflammatory activity than that of SJFE because of the higher concentration of sophoricoside relative to sophorabioside in SJSBE. LOX catalyzes the oxygenation of polyunsaturated fatty acids, which are associated with inflammation [30, 31]. Sophoricoside may inhibit the production of inflammatory mediators such as prostaglandin E2 and nitric oxide more than sophorabioside. However, further *in vivo* assays are required to validate the biological efficacy and potential application of the findings.

In conclusion, this study demonstrated the effectiveness of the biotransformation of sophorabioside into sophoricoside using α-L-rhamnosidase from *C. aurantiacus*. Optimal conditions for the enzymatic reaction were identified, with pH 6.0 and 55°C yielding the highest conversion efficiency. Methanol at 2.5% (v/v) enhanced enzymatic activity; therefore, is a suitable solvent. Substrate specificity assays revealed strong enzymatic activity on neohesperidose-containing flavonoids, including sophorabioside. The biotransformation increased the bioactive sophoricoside content in SJFE and SJSBE. Additionally, the enriched extracts exhibited significant anti-inflammatory activity, highlighting their potential for use as improved functional food supplements, providing a basis for the utilization of enzymatic biotransformation in producing high-value bioactive compounds from natural resources. These results not only demonstrate the biofunctional improvement of *S. japonica* extracts, but also highlight the potential of this enzymatic approach for industrial application, owing to its high conversion yield, mild processing conditions, and reliance on low-cost plant materials. Moreover, the enzyme exhibits favorable thermal stability (t_1/2_ = 32.2 h at 55°C), and the reaction system avoids the need for costly co-factors or complex purification, enhancing economic feasibility. These features support the commercial applicability of this process in the development of functional ingredients or nutraceutical products.

## Figures and Tables

**Table 1:** Deactivation constant (k_d_) and half-lives (t_1/2_) of α-L-rhamnosidase from *C. aurantiacus* at 45, 50, 55, 60, and 65°C.

Temperature (°C)	k_d_ (h^−1^)	t_1/2_ (h)
45	4.20 × 10^−3^	164.9
50	1.11 × 10^−2^	62.7
55	2.16 × 10^−2^	32.2
60	4.58 × 10^−2^	15.1
65	8.88 × 10^−2^	7.8

**Table 2:** Anti-inflammatory activities of SJFE, SJSBE, and biotransformed SJFE and SJSBE.

Substance	LOX inhibitory activity (%)
NDGA	67.25 ± 3.18
Baicalein	45.61 ± 2.01
Sophorabioside	22.08 ± 1.51
Sophoricoside	49.09 ± 4.13
SJFE	29.52 ± 1.66
SJSBE	38.30 ± 0.97
Biotransformed SJFE	51.85 ± 3.55
Biotransformed SJSBE	53.09 ± 1.25

Values are expressed as mean ± standard deviation (*n* = 3).
